# Management of Genetic Diversity in the Era of Genomics

**DOI:** 10.3389/fgene.2020.00880

**Published:** 2020-08-13

**Authors:** Theo H. E. Meuwissen, Anna K. Sonesson, Gebreyohans Gebregiwergis, John A. Woolliams

**Affiliations:** ^1^Department of Animal and Aquacultural Sciences, Norwegian University of Life Sciences, Ås, Norway; ^2^NOFIMA, Ås, Norway; ^3^The Roslin Institute and R(D)SVS, The University of Edinburgh, Edinburgh, United Kingdom

**Keywords:** inbreeding, genetic drift, optimum contribution selection, genetic diversity, genomic relationships, genetic gain

## Abstract

Management of genetic diversity aims to (i) maintain heterozygosity, which ameliorates inbreeding depression and loss of genetic variation at loci that may become of importance in the future; and (ii) avoid genetic drift, which prevents deleterious recessives (e.g., rare disease alleles) from drifting to high frequency, and prevents random drift of (functional) traits. In the genomics era, genomics data allow for many alternative measures of inbreeding and genomic relationships. Genomic relationships/inbreeding can be classified into (i) homozygosity/heterozygosity based (e.g., molecular kinship matrix); (ii) genetic drift-based, i.e., changes of allele frequencies; or (iii) IBD-based, i.e., SNPs are used in linkage analyses to identify IBD segments. Here, alternative measures of inbreeding/relationship were used to manage genetic diversity in genomic optimal contribution (GOC) selection schemes. Contrary to classic inbreeding theory, it was found that drift and homozygosity-based inbreeding could differ substantially in GOC schemes unless diversity management was based upon IBD. When using a homozygosity-based measure of relationship, the inbreeding management resulted in allele frequency changes toward 0.5 giving a low rate of increase in homozygosity for the panel used for management, but not for unmanaged neutral loci, at the expense of a high genetic drift. When genomic relationship matrices were based on drift, following VanRaden and as in GCTA, drift was low at the expense of a high rate of increase in homozygosity. The use of IBD-based relationship matrices for inbreeding management limited both drift and the homozygosity-based rate of inbreeding to their target values. Genetic improvement per percent of inbreeding was highest when GOC used IBD-based relationships irrespective of the inbreeding measure used. Genomic relationships based on runs of homozygosity resulted in very high initial improvement per percent of inbreeding, but also in substantial discrepancies between drift and homozygosity-based rates of inbreeding, and resulted in a drift that exceeded its target value. The discrepancy between drift and homozygosity-based rates of inbreeding was caused by a covariance between initial allele frequency and the subsequent change in frequency, which becomes stronger when using data from whole genome sequence.

## Background

Management of genetic diversity is usually directed at maintaining the diversity that was present in some population, which serves as a reference point against which diversity in the future is compared. This reference population may be some population in the past or the current population. In the absence of genomic data, the accumulated change in diversity was predicted to be a loss, and could only be described by inbreeding coefficients (*F*) based on pedigree data. These coefficients are the expectations of the loss in genetic variance relative to the reference population in which all alleles are assumed to be drawn at random with replacement, i.e., the classical base population. This description as a loss of variance is strictly for additive traits, but individual allele frequency at a locus among individuals (i.e., 0, ½, 1) is an additive trait. In this perspective, the management of genetic diversity comes down to the management of inbreeding, in particular controlling the rate of inbreeding (Δ*F*), or, equivalently, the effective population size: *N*_*e*_ = 1/(2Δ*F*) (Falconer and Mackay, 1996).

Optimal management of inbreeding in breeding schemes is achieved by optimal contribution (OC) selection (Meuwissen, 1997; Woolliams et al., 2015) that, by construction, maximizes the genetic gain made for a given rate of inbreeding. In the era of genomics, Sonesson et al. (2012) concluded that genomic selection requires genomic control of inbreeding, i.e., genomic optimal contribution selection (GOC). With OC, the management of diversity within the population uses the form 12cA′c where **A** is wright’s numerator relationship matrix and **c** is a set of fractional contributions of candidates to the next generation, and with GOC a genomic relationship matrix **G** replaces **A**. This has direct correspondence with the substantial literature on the use of similarity matrices and the fractional contributions of species as measures of species diversity (e.g., Leinster and Cobbold, 2012). The similarity matrices in OC use the idea of relationships, which are the scaled (co)variances of breeding values between all pairs of individuals in a population past and present, which links to the wider canon of genetic theory.

In the pre-genomics era, relationships were based on pedigree and pedigree-based coefficients of kinship describing the probability of identity-by-descent (IBD) at neutral loci that are unlinked to any loci under selection. Within this subset of loci, IBD results in a redistribution of genotype frequencies away from Hardy-Weinberg proportions toward homozygosity by $p_{0}^{2}\left(1-F\right)+p_{0}F,2p_{0}\left(1-p_{0}\right)\left(1-F\right)$, and (1 − *p*_0_)^2^(1 − *F*) + (1 − *p*_0_) *F* for the genotypes AA, Aa and aa, respectively, where *p*_0_ is the original frequency of the A allele (Falconer and Mackay, 1996). This redistribution of genotype frequencies links the changes of heterozygosity [expected to reduce by a factor (1–F)], the within line genetic variance [also reducing by (1–F)], and the genetic drift variance of allele frequencies [*p*_0_(1–*p*_0_)F] to the inbreeding coefficient describing the IBD of sampled alleles. These expected changes do not hold for loci linked to the causal variants of complex traits (QTL), where allele frequencies and genotype frequencies may change non-randomly, and cannot be explained by IBD predicted by pedigree alone.

When defining inbreeding as the correlation between uniting gametes, Wright (1922) assumed the infinitesimal model, which implies infinitesimal selection pressures with random changes in allele frequency. However, the genome is of finite size, and for complex traits with many QTL selection pressures will extend to neutral loci in linkage disequilibrium (LD) across the genome, and these associations to loci under selection result in non-random changes of allele frequencies. This is particularly the case for genomic selection schemes, where marker panels are large, but not infinitely large, dense and genome-wide, and designed to be in LD with all QTL, and where selection is directly for the markers included in the panel. In this setting unlinked neutral loci are likely to be rare, so the classical theory appears redundant.

Despite the apparent loss of a unifying paradigm, genomics opens up a choice of tools that could be used to describe genetic diversity that is wider in scope than the classical genetic variance and inbreeding. For example, tools based on genomic relationships (VanRaden, 2008), runs of homozygosity (de Cara et al., 2013; Luan et al., 2014; Rodríguez-Ramilo et al., 2015), and linkage analysis (Fernando and Grossman, 1989; Meuwissen et al., 2011). Some genomic measures may be better suited for some purposes than others, and so the question arises of what is the purpose of the management of diversity in breeding schemes in addition to what tools to use. Furthermore, when considering tools for genomic inbreeding, there is a need to distinguish which aspect of inbreeding they depict (IBD, heterozygosity/homozygosity, or genetic drift), since in (genomic) selection schemes their expectations may differ from those derived from random allele frequency changes resulting in the genotype frequencies $p_{0}^{2}\left(1-F\right)+Fp_{0},2p_{0}\left(1-p_{0}\right)\left(1-F\right)$, and (1 − *p*_0_)^2^(1 − *F*) + *F*(1 − *p*_0_).

Most molecular genetic measures of inbreeding are based on the allelic identity of marker loci, and do not directly separate IBD from Identity-By-State (IBS). Genomic relationship matrices which are variants of VanRaden (2008) compensate for this by measuring squared changes in allele frequency relative to a set of reference frequencies. For the purposes of managing changes in diversity relative to the reference population these frequencies would be those relevant to this base generation (Sonesson et al., 2012), although often the frequencies in the current “generation” are used (Powell et al., 2010), or simply the subset of the population for which the genomic data is available; see Legarra (2016) for further discussion on these issues. Providing the base generation is used to define the reference frequencies at neutral unlinked loci (*p*_0,*k*_ for locus k), the expectation of **G**_VR2_ (Method 2; VanRaden, 2008) is **A**, with all loci equally weighted after standardization using the base generation frequencies. In comparison, **G**_*VR1*_ (Method 1) can be viewed as simply re-weighting the loci by 2*p*_0,*k*_(1−*p*_0,*k*_): i.e., for a single locus, **G**_*VR1*_ and **G**_VR2_ yield identical relationship estimates, and extending to many loci **G**_*VR*2_ uses the simple mean of the single locus estimates whereas **G**_*VR*1_ uses the weighted mean with 2*p*_0,*k*_(1−*p*_0,*k*_) as the weights. Extending the argument of Woolliams et al. (2015) for **G**_*VR1*_, since **G**_VR2_ is based on the squares of standardized allele frequency changes, and the management of diversity using **G**_VR2_ will constrain these squared standardized changes; this measurement of inbreeding will be denoted as *F*_drift_ [see Eq. (1B) in Methods section for a more precise definition]. When using 0.5 as the base frequency for all loci, as sometimes proposed, the relationship matrix *G*_*VR*_0.5__ is proportional to homozygosity and molecular coancestry (Toro et al., 2014). Hence, *G*_VR_0.5__ may be used to measure homozygosity-based inbreeding, *F*_hom_, and the loss of heterozygosity (1–*F*_hom_).

The use of a genomic relationship matrix, **G**_LA_, based on linkage analysis for inbreeding management was suggested and studied by Toro et al. (1998), Wang (2001), Pong-Wong and Woolliams (2007), Fernandez et al. (2005), and Villanueva et al. (2005). Here the inheritance of the marker alleles is used to determine probabilities of having inheriting the maternal or paternal allele from a parent at the marker loci instead of assuming 50/50 inheritance probabilities as in **A**. **G**_LA_ thus requires pedigree and marker information, and IBD relationships are relative to the (assumed) unrelated and non-inbred base population as in **A**. In this way IBD is evaluated directly by **G**_LA_, and is not simply an expectation for neutral unlinked loci as described above for **G**_VR2_. If two (base) individuals are unrelated in **A** then they are unrelated in **G**_LA_, whereas the other measures also estimate (non-zero) relationships for base population individuals. The marker data accounts for Mendelian segregation which may deviate from 50/50 probabilities through any linkage drag from loci under selection, or selective advantage. **G**_LA_ can be constructed by a tabular method, similar to that for the pedigree based relationship matrix (Fernando and Grossman, 1989), and software for the simultaneous linkage analysis of an entire chromosome is available (e.g., LDMIP (Linkage Disequilibrium Multilocus Iterative Peeling); Meuwissen and Goddard, 2010). **G**_LA_ is a tool that specifically describes IBD across the genome, hence we will denote this IBD based estimate of inbreeding as *F*_IBD_.

A run of homozygosity (ROH) is an uninterrupted sequence of homozygous markers (McQuillan et al., 2008). The exact definition of a ROH differs among studies as a number of ancillary constraints are added related to the minimum length of a ROH measured in markers and/or cM, minimum marker density, and in some cases an allowance for some heterozygous genotypes arising from genotyping errors. The idea is that a run of homozygous markers indicates an IBD segment, since it is unlikely that many consecutive homozygous markers are IBS by chance alone. The total length of ROH relative to the total genome length provides an estimate of *F*_IBD_ from the DNA itself, and this estimate will be denoted *F*_ROH_. The reference population for *F*_ROH_ is unclear, although by varying the constraint on the length of the ROHs the emphasis can be changed from old inbreeding, with short ROHs, to young inbreeding, with long ROHs (Keller et al., 2011). *F*_ROH_ may miss some relevant inbreeding since IBD segments shorter than the minimum length are neglected. On the one hand, *F*_ROH_ is an IBD based measure of inbreeding, as it attempts to identify IBD segments (especially when ROHs are long), but on the other hand it is a homozygosity based measure of inbreeding since it is actually based on the homozygosity of haplotypes (especially when ROHs are short). However, *F*_ROH_ is a measure of inbreeding in a single individual and is unsuitable for a measure of IBD within the population as a whole. Therefore integration of ROH into a GOC framework requires a pairwise measurement to form a similarity matrix, **G**_ROH_ (de Cara et al., 2013).

The aim of this study is to: (i) re-examine the goals of the management of genetic diversity in breeding schemes, and the molecular genetic parameters that may be incorporated into these goals; and (ii) compare alternative genomic- and pedigree-based measures of inbreeding and relationships for addressing the goals. In doing so the different tools discussed above and some novel variants will be compared for their ability to generate gain in breeding schemes while measures of inbreeding are constrained. Finally, conclusions are made with respect to the practical implementation of these tools for managing diversity and how the outcomes will depend on whether whole genome sequence (WGS) data is considered or marker panels.

## Materials and Methods

### The Goals of the Management of Genetic Diversity

Managed populations, such as livestock, will generally have many desirable characteristics (related to production, reproduction, disease resistance, etc.). Some of these characteristics are to be improved (the breeding goal traits), without jeopardizing the others. The latter is the aim of the management of inbreeding. Specifically, breeding programs aim to change allele frequencies at the QTL in the desired direction. This ultimately results in loss of variation at the QTL as fixation approaches, but providing these changes are in the right direction this loss of variation is not a problem. However, genetic drift from our reference population and loss of variation at loci that are neutral for the selection goal are to be avoided for the following reasons. Firstly, to alleviate the risk of inbreeding depression through decreased heterozygosity, particularly for traits that are not under artificial selection but are needed for the healthy functioning of the animals. Secondly, deleterious recessive alleles may drift to high frequencies, and occur more frequently in their deleterious or lethal homozygous form; although mentioned separately this is a specific manifestation of inbreeding depression. In the genomics era, deleterious recessives may be identified and mapped (Charlier et al., 2008), and if achieved recessive mutations may be selected against (at the cost of selection pressures), or potentially gene-edited. Nonetheless, simultaneous selection against many genetic defects diverts substantial selection pressures away from other traits in the breeding goal. Thirdly, loss of variation arising from selection sweeps for the current goal may erase variation for traits that are currently not of interest but may be valued in the future and so limit the future selection opportunities. Fourthly, genetic drift in the sense of random changes of allele frequencies, and thus random changes of trait values, which may be deleterious. This encompasses both the traits outside the current breeding goal and within it, where drift is observed as variability in the selection response. Moreover, large random changes in allele frequency may disrupt positive additive-by-additive interactions between QTL which have occurred due to many generations of natural and/or artificial selection (similar to recombination losses in crossbreeding; Kinghorn, 1980). In addition, random allele frequency changes may result in the loss of rare alleles, which implies a permanent loss of variation.

### Measures for Management of Inbreeding

Whilst genomics offers molecular measures for direct monitoring, most obviously heterozygosity and frequency changes measured from a panel of anonymous markers, the strategy for management of these diverse problems using genomics does not follow directly. For example, increasing heterozygosity *per se*, achieved by moving allele frequencies of marker loci toward ½ is not solely beneficial, as while potentially ameliorating the aforementioned problems 1 and 3 it is deleterious for problems 2 and 4. Both these empirical measures of heterozygosity and the change of frequencies from drift can be considered to be measures of inbreeding and diversity. Wright (1922) states that a natural inbreeding coefficient moves between 0 and 1 as heterozygosity with random mating moves between its initial state and 0: therefore, if a locus *k* has initial frequency *p*_0_ and current frequency *p*_*t,k*_ then a measure of inbreeding is 1−(*H*_*t*,*k*_/*H*_0,*k*_) = 1−[2*p*_*t*,*k*_(1−*p*_*t*,*k*_)]/[2*p*_0,*k*_(1−*p*_0,*k*_)], which can be generalized by averaging loci to obtain *F*_hom_, i.e.,

(1A) $$F_{\text{hom}}=1-\sum_{\text{loci}k}\frac{2p_{t,k}\left(1-p_{t,k}\right)}{2p_{0,k}\left(1-p_{0,k}\right)}/N_{\text{SNP}}$$

where N_*SNP*_ is the total number of loci. *F*_hom_ can be negative when heterozygosity increases due to allele frequencies moving toward 0.5. Similarly, drift can be measured as $\delta p_{t,k}^{2}={\left(p_{t,k}-p_{0,k}\right)}^{2}$, scaled by the expected value for complete random inbreeding, i.e., $\delta p_{t,k}^{2}/\left[p_{0,k}\left(1-p_{0,k}\right)\right]$, and similarly averaged over loci to obtain *F*_drift_, i.e.,

(1B) $$F_{\text{drift}}=\sum_{\text{loci}k}\frac{\delta p_{t,k}^{2}}{p_{0,k}\left(1-p_{0,k}\right)}/N_{\text{SNP}}$$

and which is never negative. *F*_drift_ is similar to the definition of *F*_*ST*_ (Holsinger and Weir, 2009), which is here applied to a single population over time instead of a sample of populations, and it is this empirical measure that is being directly addressed when using *G*_*VR*2_.

For locus *k* in the set of neutral loci with frequency *p*_0,*k*_ in the base population and frequency *p*_*t*,*k*_ = *p*_0,*k*_ + δ*p*_*t*,*k*_ in generation t, twice the frequency in generation t is $2p_{t,k}^{2}+H_{t,k}=2\left(p_{0}+\delta p_{t,k}\right)$, where *H*_*t*,*k*_ = 2(*p*_0_ + δ*p*_*t*,*k*_)(1−*p*_0_−δ*p*_*t*,*k*_), which holds for all loci assuming random mating. With a sufficiently large subset of neutral loci with the same base frequency *p*_0_ if *E*[δ*p*_*t*,*k*_|*p*_0_] = 0 then taking expectations over this subset $2E\left[p_{t,k}^{2}\right]+E\left[H_{t,k}\right]=2p_{0}$ and so $2\left(E\left[p_{t,k}^{2}\right]-p_{0}^{2}\right)+E\left[H_{t,k}\right]=2p_{0}\left(1-p_{0}\right)$. The first term is 2*var*(*p*_*t*,*k*_) and the second is *H*_*t*_ and dividing through by 2*p*_0_(1−*p*_0_) gives

(2) $$var\left(p_{t,k}\right)/\left[p_{0}\left(1-p_{0}\right)\right]=1-H_{t,k}/H_{0}\;\Rightarrow \;F_{\text{drift}}=F_{\text{hom}}$$

Therefore if *E*[δ*p*_*t*,*k*_|*p*_0_] = 0 over the range 0 < *p*_0_ < 1, there is an equivalence of *F*_drift_ with *F*_hom_ irrespective of initial frequency, *p*_0_ (Falconer and Mackay, 1996): i.e., drift- and homozygosity-based inbreeding are expected to be the same if allele frequency changes are on average 0 irrespective of the initial frequency.

Using a form of GOC related to *G*_*VR1*_ (see Discussion), de Beukelaer et al. (2017) explore the management of diversity and derived the consequences for the rate of homozygosity, $2\left(\delta p_{t,k}^{2}+2\delta p_{t,k}\left(p_{0}-\frac{1}{2}\right)\right)/H_{t,k}$. They suggested (supported by results below) that the term $\delta p_{t,k}\left(p_{0}-\frac{1}{2}\right)$, which represents a covariance between allele frequency change δ*p*_*t*,*k*_ and initial frequency *p*_0,*k*_ across the loci *k*, may be non-zero. Consequently, *E*[δ*p*_*t*,*k*_|*p*_0_]≠0, and Equation [2] will no longer hold, and *F*_drift_≠*F*_hom_. Supplementary Information 1 shows that *any* deviation from Equation [2] for a general set of loci for which *E*[δ*p*_*t*,*k*_] = 0 over the set, not necessarily with the same initial frequency, must be explained by a covariance between allele frequency changes and the original frequency cov(δ*p*_*t*,*k*_;*p*_0,*k*_) and shows:

(3) $$\begin{array}{c} F_{\text{hom}}-F_{\text{drift}}=2\text{cov}(\delta p_{t,k}/\sqrt{p_{0,k}\left(1-p_{0,k}\right)};\\ \left(p_{0,k^{-1/2}}\right)/\sqrt{p_{0,k}\left(1-p_{0,k}\right)}) \end{array}$$

i.e., if there is covariance between initial allele frequencies and frequency changes, homozygosity and drift based inbreeding are no longer equal. Therefore this covariance will be important in determining the impact of genomic management, which aims to manage both the increase of homozygosity and genetic drift.

Supplementary Information 1 explores why completely random selection of parents (i.e., with no management) generates no covariance and how different broad management goals for diversity may generate a covariances of different signs. In particular, with completely random selection, most markers drift to the nearest extreme with the smaller change in frequency, but a minority will move to the opposite extreme resulting in the larger frequency change, giving a net result of no covariance. The consequence of using GOC based on *G*_*VR2*_ is that the latter large allele frequency changes are penalized more heavily, since they add as $\delta p_{t,k}^{2}$ to the elements of *G*_*VR2*_ and consequently to 12cG′c. Hence, the hypothesis is tested below that *G*_*VR2*_ emphasizes the movement of MAF toward 0, and more generally allele frequencies move away from intermediate values toward the nearest extreme, resulting in *cov*(δ*p*_*t*,*k*_;*p*_0,*k*_) > 0 and *var*(*p*_*t*,*k*_)/[*p*_0_(1−*p*_0_)] + *E*[*H*_*t*,*k*_/*H*_0,*k*_] < 1, contrary to expectations in Eq. (2).

Conversely if *G*_0.5_ is used in GOC then there will be pressure to move allele-frequencies toward 0.5 resulting in increasing heterozygosity (Li and Horvitz, 1953). Supplementary Information 1 shows that this results in *cov*(δ*p*_*t*,*k*_;*p*_0,*k*_) < 0, and thus *F*_hom_ < 0, and *F*_drift_ > 0, and *var*(*p*_*t*,*k*_)/[*p*_0_(1−*p*_0_)] + *E*[*H*_*t*,*k*_/*H*_0,*k*_] > 1, again contrary to expectations in Eq. (2). Furthermore the implication of these considerations is that the covariance *cov*(δ*p*_*t*,*k*_;*p*_0,*k*_) is a property of the active management of diversity using squared frequency changes as in *G*_*VR2*_ (or *G*_*VR1*_) and not as a consequence of directional selection. This hypothesis was tested below in two ways: firstly by combining the management of diversity using *G*_*VR2*_ with randomly generated EBVs, and secondly by using a panel of markers for managing diversity that is distinct from the panel used for estimating GEBVs for genomic selection.

The term $\delta p_{t,k}^{2}/\left[p_{0,k}\left(1-p_{0,k}\right)\right]$ appearing in *F*_drift_ can be viewed as an approximation to the squared total intensity (*i*^2^) applied to the marker, where *i*≈δ*p*_*t*,*k*_/[*p*_0,*k*_(1−*p*_0,*k*_)]. The approximation arises because the total selection intensity applied to a marker is not linear with frequency (see Liu and Woolliams, 2010). For example, after the initial generation, the intensity applied to alleles moved toward ½ is overestimated, since the denominator of *i* increases over time, which reduces the actual intensity applied. The opposite holds for those alleles moved toward the nearest extreme. Therefore a further hypothesis is that a relationship matrix built upon *i*^2^, *G*_*i(p)*_, rather than $\delta p_{t,k}^{2}$ may remove the covariance of the change in frequency with the initial frequency that is generated using *G*_*VR2*_. More details on this and the calculation of *G*_*i(p)*_ are given in Supplementary Information 2.

In classical theory, the equivalence of *F*_drift_ with *F*_hom_ under random mating is an outcome of considering IBD, and management by IBD. The genomic relationship matrices based on allele frequency changes or functions of these changes no longer consider IBD as they only consider IBS. Supplementary Information 3 considers the IBD properties of the linkage analysis relationship matrix *G_LA_* which is derived from the markers. Considering the management of diversity over generations when using *G_LA_*, the conclusion of Supplementary Information 3 is that δ*p*_*t*,*k*_ will now be determined by the properties of the base population and not through linkage disequilibrium generated in the course of the selection process. Therefore, the covariance between the change in frequency and its initial value is potentially avoided. This leads to a further hypothesis tested below that if *G*_*LA*_ replaces *G*_*VR2*_ in GOC then *F*_drift_ = *F*_hom_ and *var*(*p*_*t*,*k*_)/[*p*_0_(1−*p*_0_)] + *E*[*H*_*t*,*k*_/*H*_0,*k*_] = 1, as expected in Eq. (2); i.e., consideration of IBD restores the equivalence of *F*_drift_ and *F*_hom_ for a set of neutral markers. If *A* or a ROH-based *G*_*ROH*_ replaces *G*_*LA*_ the same hypothesis may be advanced given their focus on approximating IBD, however, both are approximations to the true genomic IBD that is tracked by *G*_*LA*_ and so the equivalence may only be approximate.

In summary, there are a range of hypotheses to be tested on three categories of relationship matrix: those based on drift, changes in allele frequency or functions of them (*G*_*VR*1_,*G*_*VR*2_,and*G*_*i*(*p*)_); those based on homozygosity exemplified by *G*_0.5_; and those based on IBD (*G*_*LA*_ and **A**). A relationship matrix based on ROH, *G*_*ROH*_, is a hybrid of the latter two, targeting IBD by measuring homozygosity of haplotypes.

### Breeding Structure and Genomic Architecture

A computer simulation study was conducted to compare these alternative GOC methods. The simulations mimicked a breeding scheme using sib-testing, such as those used for disease challenges in fish breeding, which is similar to Sonesson et al. (2012). The scheme had a nucleus where selection of candidates was entirely based on their genomic data and performance recording was solely on the full-sibs of the selection candidates which were also genotyped. This scheme may be considered extreme in the sense that the candidates themselves have no performance records, and is practiced in aquaculture to prevent disease infections within the breeding population. There were 2000 young fish per generation, and every full-sib family was split in two: half of the sibs became selection candidates and the other half test-sibs. The actual number of families and their size depended on the optimal contributions of the parents.

The genome consisted of 10 chromosomes of size 1 Morgan. Base population genomes were simulated for a population of an effective size of *N*_*e*_ = 100 for 400 (=4*N*_*e*_) generations with SNP mutations occurring at a rate of 10^–8^ per base pair per generation using the infinite-sites model. This resulted in WGS data for base population genomes that were in mutation-drift-linkage disequilibrium balance. The historical population size was chosen to equal the effective population size targeted for the breeding schemes and so avoid any effect of a sudden large change in effective population size. This resulted in 33,129 segregating SNP loci, which is relatively small in number due to the small effective size of 100. From these loci *N*_SNP_ = 7000 were randomly sampled as marker loci for use in obtaining GEBV by genomic selection (Panel M); another distinct sample of 7000 loci were randomly sampled as additive QTL, which obtained an allelic effect sampled from the Normal distribution (Panel Q); and a further distinct sample of 7000 SNP loci were randomly sampled to act as “neutral loci” (Panel N), which were used to assess allele-frequency changes and loss of heterozygosity at neutral (anonymous) WGS loci, not involved in either genomic prediction or diversity management. In the majority of schemes Panel M was used for constructing genomic relationship matrices for both obtaining EBVs and diversity management. However, to test whether the non-neutrality of the SNPs used for genomic prediction interfered with their simultaneous use for diversity management, a further distinct panel of 7000 randomly picked loci (Panel D) was used for diversity management in some schemes.

True breeding values were obtained by summing the effects of the QTL alleles across the loci in Panel Q, before scaling them such that the total genetic variance was $\sigma_{g}^{2}=1$ in the base population. Phenotypes were obtained by adding a randomly sampled environmental effect with variance $\sigma_{e}^{2}=1.5$, resulting in a heritability of 0.4. After the initial 400 unselected generations to simulate a base population (*t* = 0), the breeding schemes described below were run for 20 generations, of which the first generation comprised random selection in order to create an initial sib-family structure.

### Genomic Estimates of Breeding Values

GEBV $\left(\hat{g}\right)$ were obtained by the SNP-BLUP method (Meuwissen et al., 2001) where BLUP estimates of SNP effects were obtained from random regression on the SNP genotypes of Panel M coded as *X*_*ik*_ = –2*p*_0,*k*_/√[2*p*_0,*k*_(1–*p*_0,*k*_)], (1–2*p*_0,*k*_)/√[2*p*_0,*k*_(1–*p*_0,*k*_)], or (2–2*p*_0,*k*_)/√[2*p*_0,*k*_(1–*p*_0,*k*_)] for homozygote, heterozygote, and alternative homozygote genotypes, respectively, of the *k*th SNP of animal *i*, and *p*_0,*k*_ is the allele frequency of a randomly chosen reference allele of the *k*th SNP in generation 0. The model for the BLUP estimation of the SNP effects was:

y=1μ+Xb+e

where ***y*** is a vector of records; μ is the overall mean; ***X*** is a matrix of genotype codes as described above; ***b*** is a vector of random SNP effects [*a priori*, $b∼MVN\left(0,\sigma_{g}^{2}N_{SNP}^{-1}I\right)$], and ***e*** is a vector of random residuals [*a priori $e∼N\left(0,\sigma_{e}^{2}I\right)$*]. GEBV were obtained as $\hat{g}=X\hat{b}$ where $\hat{b}$ denotes the BLUP estimates of the SNP effects. This model is often implemented in the form of GBLUP using VanRaden (2008) Model 2, which assumes that all loci explain an equal proportion of the genetic variance. When simulating true breeding values, variances of allelic effects were equal across the loci, which implies that the high-MAF QTL explain more variance than the low-MAF QTL. Hence, there is a discrepancy between the simulation model and that used for analysis. However, such discrepancies always occur with real data. To separate the effects of selection and inbreeding management, one of the schemes described below randomly sampled GEBVs from a Normal distribution each generation.

### Assessing the Rates of Inbreeding at Neutral Loci

*F*_hom_ and *F*_drift_were calculated for each scheme, and since discrepancies were anticipated (Supplementary Information 1) Δ*F*was also calculated from both heterozygosity and drift to give Δ*F*_hom_ and Δ*F*_drift_. The calculations described below were done for all schemes with Panel N which were both functionally neutral in not influencing the breeding goal traits, and algorithmically neutral in not being involved in the breeding value prediction. Calculations were repeated for Panel M, and Panel D when used.

#### Heterozygosity

Calculation was based upon classical models where for generation *t* (Σ_loci*k*_*H*_*t*,*k*_/*H*_0,*k*_)/*N*_SNP_ = 1−*F*_hom_ = (1−Δ*F*)^*t*^ where Δ*F* is the rate of inbreeding, and *N*_*SNP*_the number of loci in the panel. A log transformation yields a linear relationship log⁡(Σ_loci*k*_*H*_*t*,*k*_/*H*_0,*k*_)−log⁡(*N*_SNP_) = *t*log⁡(1−Δ*F*)≈−*t*Δ*F*, where the approximation holds for small Δ*F* when using natural logarithms. This regression was calculated and provided both a test of constant Δ*F*_hom_ and an estimate of Δ*F*_hom_ from (−1) × slope of the regression.

#### Drift

At time *t*,*F*_drift_ was calculated as Σ_loci*k*_(*p*_*t*,*k*_−*p*_0,*k*_)^2^/[*p*_0,*k*_(1−*p*_0,*k*_)]. Analogously with heterozygosity, classical theory was followed by taking logs of (1−*F*_drift_) with Δ*F*_drift_estimated by −1 × slope from the regression on *t*.

### Optimum Contribution Selection Methods

In optimal contribution selection, the rate of inbreeding is constrained by constraining the increase of the group coancestry of the selected parents, $\bar{G}=\frac{1}{2}c^{\prime }Gc$, where *G* denotes the relationship matrix of interest for managing diversity among the selection candidates, and **c** denotes a vector of contributions of the selection candidates to the next generation, which is proportional to their numbers of offspring. Therefore the group coancestry is the average relationship among all pairs of the parents, including self-pairings, weighted by the fraction of offspring from the pair assuming completely random mating. Furthermore, the genetic level of the selected animals, $\bar{g}=c^{\prime }\hat{g}$, is maximized weighted by their number of offspring. Hence, the optimisation is as follows:

$$\text{maximize}\;\bar{\text{g}}={\text{c}}^{\prime }\hat{\text{g}}\mathrm{by}\mathrm{varying}\text{c}$$

$$\mathrm{with}\mathrm{constraints}:\;K=\frac{1}{2}c^{\prime }\mathrm{Gc}$$

$$\Sigma_{j\text{males}}c_{j}=\frac{1}{2}$$

$$\Sigma_{j\text{females}}c_{j}=\frac{1}{2}$$

$$c_{j}\geq 0\mathrm{for}\mathrm{all}j.$$

A number of relationship matrices were investigated for managing the diversity: (i) the pedigree-based relationship matrix **A**; (ii) the genomic relationship matrix **G**_*VR*2_ = *XX*′/*N*_SNP_ (VanRaden, 2008; Model 2) constructed using Panel M; (iii) the genomic relationship matrix *G*_*VR*1_ = *ZZ*′/Σ_loci*k*_*H*_0,*k*_ (VanRaden, 2008; Model 1) constructed using SNP Panel M where *Z*_*ij*_ = (−2*p*_0*j*_),(1−2*p*_0*j*_),or(2−2*p*_0*j*_); (iv) *G*_0.5_, a homozygosity based matrix of relationships, since its elements (*i,j*) are proportional to the expected homozygosity of progeny of animals *i* and *j* (Toro et al., 2014); (v) *G*_*LA*_constructed from Panel M using linkage analysis (Fernando and Grossman, 1989; Meuwissen et al., 2011); (vi) a novel relationship matrix *G*_*i(p)*_ constructed from squared total applied intensities using Panel M (see Supplementary Information 2); (vii) the genomic relationship matrix *G*_*ROH*_ based on ROH assessed using Panel M following the method of de Cara et al. (2013) (see Supplementary Information 2); (viii) a genomic relationship matrix *G*_VR2_ constructed using Panel D instead of M. In this replicated simulation study, the calculation of *G*_LA_ by LDMIP (Meuwissen and Goddard, 2010) was computationally too demanding and instead, a haplotype-based approach was adopted as an approximation (see Supplementary Information 2).

### Implementation of Selection Procedures

The selection schemes simulated will be denoted by the relationship matrix used in GOC and the panel of markers used for SNP-BLUP and building the relationship matrix. The panel for SNP-BLUP was either “M”, or “∼” when using randomly generated GEBV. The latter implements a scheme without directional selection, and tests whether observed results are due to selection or due to diversity management. The panel for management of inbreeding was either “M,” “D,” or “∼” when using **A** which required no marker panel. Therefore a total of 9 schemes contribute to the results presented: 6 of which are of the form **G**(M,M) where **G** is either **G**_*VR*1_, **G**_VR2_, **G**_0.5_, **G**_LA_, **G**_*i(p)*_, and **G**_ROH_; with the remaining three being **A**(M,∼), **G**_VR2_(M,D), and **G**_VR2_(∼,M), where the first symbol in parentheses refers to EBV estimation and the second to diversity management. The schemes are summarized in Table 1.

**TABLE 1:** The relationship matrices and marker panels that were used for the alternative breeding schemes.

	Marker Panel^1^		F-management	
Scheme^2^	EBV-estimation	F-management	Matrix^3^	Type of measure
G_VR2_(M,M)	M	M	G_VR2_	Drift
G_VR2_(M,D)	M	D	G_VR2_	Drift
G_VR2_(∼,M)	∼	M	G_VR2_	Drift
G_*VR1*_(M,M)	M	M	G_*VR1*_	Drift
G_*i(p)*_(M,M)	M	M	G_*i(p)*_	Drift
G_0.5_(M,M)	M	M	G_0.5_	Homoz.
G_ROH_(M,M)	M	M	G_ROH_	Homoz.
G_LA_(M,M)	M	M	G_LA_	IBD
A(M,∼)	M	∼	A	IBD

*^1^M = regular marker panel used for selection (and management); D = an extra marker panel solely used for inbreeding management (if used at all); ∼ = no markers needed for management / selection (∼ for selection implies random selection). ^2^Breeding schemes are denoted by G(P_EBV_,P_F_) where G denotes the relationship matrix used for inbreeding management (all schemes used **G**_VR2_ for EBV estimation); P_EBV_ denotes the marker panel used for EBV estimation; and P_F_ denotes the marker panel used for inbreeding management. ^3^Abbreviations and calculations of the relationship matrices are explained in the main text.*

For all schemes the target Δ*F* was set via the parameter *K* to 0.005 / generation, so the target effective population size was 100. Therefore the group coancestry of the parents was set in generation *t* to *K*_*t*_ = *K*_*t*−1_ + 0.005(1−*K*_*t*−1_), where $K_{0}=1/2\bar{G}$ and $\bar{G}$ denotes the average relationship of all candidates in generation 1 (the first generation with GOC selection). Each scheme was replicated 100 times by generating a new base population as described above. Simulation errors were reduced by simulating all alternative breeding schemes on each replicate of the initial generations, using the same Panels M, Q, N, and D, and the same effects for the QTLs. Each generation had random mating among males and females with mating proportions guided by the optimum contributions **c**.

**G**_LA_ and **A** are mathematically guaranteed to be positive definite, and **G**_*VR*1_, **G**_VR2_, **G**_0.5_, and **G**_*i(p)*_ are guaranteed to be positive semi-definite, i.e., all eigenvalues λ_*i*_≥0, as they are the cross-product of SNP genotype matrices (**X** or **Z**) with one eigenvalue of zero due to the centring of the genotypes. For the semi-definite matrices a small value (α = 0.01) was added to their leading diagonal to make them invertible, and positive definite to permit the use of the optimal contribution algorithm of Meuwissen (1997). In contrast, **G**_ROH_ is not guaranteed to be semi-positive definite since its elements are calculated one by one, and large negative eigenvalues for **G**_ROH_ were observed empirically (results not shown). When using a general matrix inversion routine the achieved Δ*F* were much larger than 0.005/generation. Hence, **G**_ROH_ was made positive definite by adding substantial values of α to its diagonals, chosen by trial and error. Starting from an initial value of α = 0.05, positive definiteness was tested by inversion using Cholesky decomposition, and if it failed then α was doubled if α < 1 or increased by 1 otherwise, until inversion was successful.

## Results

### SNPs

**FIGURE 1:**
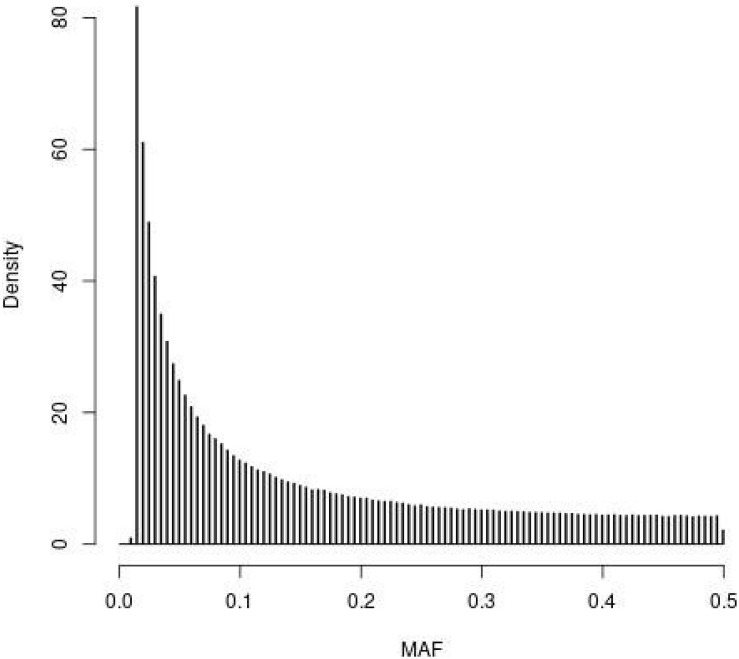
Histogram of the minor allele frequencies (MAF) of the SNPs in the whole genome sequence of the founder population (*t* = 0) observed in the simulations following 4000 generations of mutation and random selection.

The distribution of MAF for the SNPs in the WGS of the founder population (*t* = 0) observed in the simulations is depicted in Figure 1. The four SNP panels, i.e., M, the SNP-BLUP panel, N, the neutral marker panel, Q, the QTL panel, and D, a second marker panel for genetic diversity management, are random samples from the SNPs depicted in Figure 1. The MAF distribution is typical for that of whole genome sequence data with very many SNPs with rare alleles and relatively few SNPs with intermediate allele frequencies.

### Equivalence of *F*_drift_ and *F*_hom_

**FIGURE 2:**
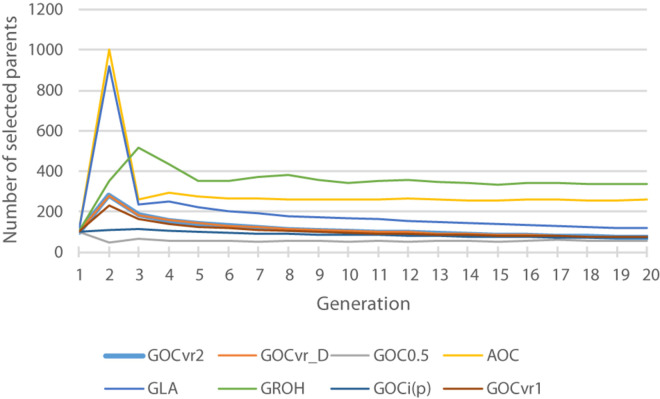
The total number of selected parents for each generation for different breeding schemes. The total is the number of animals with optimal contributions >0 required to achieve a fractional increase in the OC constraint of 0.005.

Table 2 shows for the alternative breeding schemes the drift- and homozygosity-based rates of inbreeding, together with the deviations *F*_hom_–*F*_drift_ in generation 20. For classical inbreeding theory the expectation is that *F*_hom_ = *F*_drift_ = 0.095 for random mating. However, with two sexes there will be deviations which depend on the number of mating parents which are shown in Figure 2 and were approximately equally divided between males and females each generation. This has an impact in decreasing F_hom_ at generation 20 below random mating expectations by approximately 1/(2T) where T is the total number of parents following Robertson (1965). Therefore at generation 20, there is a classical expectation for F_drift_ to exceed F_hom_ by ∼0.001 for schemes **G**_ROH_(M,M) and **A**(M,∼), through ∼0.005 for **G**_LA_(M,M) to ∼0.01 for **G**_VR2_(M,M).

**TABLE 2:** Rates of increase of homozygosity (Δ*F*_hom_), drift (Δ*F*_drift_), and the deviation *F*_hom_–*F*_drift_ in generation 20 for different types of diversity measures for Panels M and N.

Scheme^1^	GBLUP loci (Panel M)			Neutral loci (Panel N)		
	Δ*F*_HOM_^2^	Δ*F*_drift_^2^	*F*_hom_–*F*_drift_^3^	Δ*F*_HOM_^2^	Δ*F*_drift_^2^	*F*_hom_–*F*_drift_^3^
**Drift measures**						
G_VR2_(M,M)	0.0146	0.005	0.147	0.0103	0.0068	0.054
G_VR2_(M,D)	0.01	0.0069	0.048	0.0101	0.0068	0.05
G_VR2_(~,M)	0.0109	0.005	0.093	0.0085	0.0059	0.041
G_*VR1*_(M,M)	0.0096	0.0056	0.063	0.008	0.0069	0.021
G_*i(p)*_(M,M)	0.0051	0.0071	−0.053	0.0065	0.0077	−0.031
**Homozygosity measures**						
G_0.5_(M,M)	0.0008	0.0213	−0.348	0.0073	0.0176	−0.17
G_ROH_(M,M)	0.0042	0.0091	−0.102	0.0054	0.0088	−0.07
**IBD measures**						
G_LA_(M,M)	0.0044	0.0049	−0.009	0.0043	0.0049	−0.01
**A**(M,~)	0.0072	0.0083	−0.016	0.007	0.0084	−0.021

*The target rate of inbreeding for the management of genetic variation was 0.005, and results weigh loci equally irrespective of initial frequency. ^1^See Table 1 for scheme names. ^2^Standard errors <2.5 × 10^–5^. ^3^Standard errors <2.2 × 10^–4^.*

The deviations of *F*_hom_–*F*_drift_ from 0 were significant for all the schemes, for both the SNP-BLUP Panel M and the neutral Panel N, and would imply significant deviations from the classical Eq. (2). The deviation *F*_hom_–*F*_drift_ for **G**_LA_(M,M) was closest to the classical expectation, and was closer still after accounting for the degree of non-random mating that was present. Among the remaining schemes **A**(M,∼) most closely aligns to classical expectations. The results based on ROH which attempts to mimic IBD appears more similar to **G**_0.5_(M,M) which manages homozygosity, where F_drift_ exceeds F_hom_, although the deviations of the **G**_0.5_(M,M) scheme are much larger, with *F*_hom_−*F*_drift_ = −0.347 for Panel M which is more than a third of the maximum inbreeding coefficient of 1.

**FIGURE 3:**
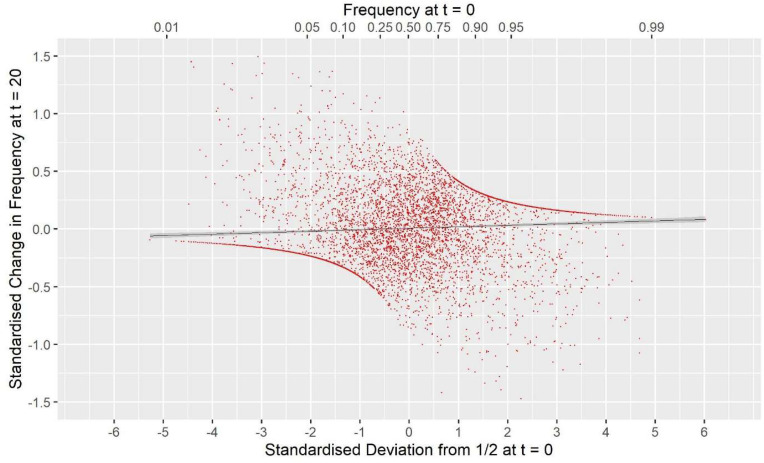
The covariance between the standardized change in allele frequency at *t* = 20 and the standardize frequency at *t* = 0 for the 7000 SNP loci in Panel N for a randomly chosen replicate. Standardization is by $\sqrt{p_{0,k}\left(1-p_{0,k}\right)}$ for locus *k*. The solid black line is the fitted linear regression *y* = 0.0083 + 0.0070×, with SES 0.0042 and 0.0021, respectively, and a Pearson correlation *r* = 0.040. For this replicate *F*drift = 0.123, *F*hom = 0.178, and twice the covariance was 0.0555. The upper *x*-axis shows the untransformed frequency.

**G**_VR2_(M,M), i.e., a commonly used GOC scheme, showed a large deviation opposite to that for **G**_0.5_(M,M) with *F*_hom_−*F*_drift_ = 0.147 for Panel M, and 0.053 for Panel N, an excess of loss of heterozygosity relative to drift. Supplementary Information 1 shows this discrepancy must arise due to a covariance between the direction of allele frequency change and initial frequency, with a stronger drift to extremes than would be expected in classical theory. Figure 3 illustrates this covariance for a randomly chosen replicate, and shows the regression line (*P* < 0.001); for this replicate the difference *F*_hom_−*F*_drift_ = 0.055 in Panel N, which arose from a correlation of only 0.040. For **G**_*VR1*_(M,M), which compared to **G**_VR2_(M,M) weights the Panel M loci proportional to 2*p*_0,*k*_(1−*p*_0,*k*_), this covariance was weaker but was still observed. The result for **G**_VR2_(M,D) showed that if the panel used for managing diversity (D) is distinct from that used for SNP-BLUP (M), the covariance in Panel M became similar to that for Panel N, as it is no longer directly managed for its diversity, and the outcome for the unmanaged neutral Panel N was almost identical to **G**_VR2_(M,M). The hypothesis that the covariance arises solely as a property of the management by **G**_VR2_, rather than as a consequence of the directional selection, was confirmed by the results for **G**_VR2_(∼,M) where F_hom_ still exceeded F_drift_. Managing the intensity in scheme **G**_*i(p)*_(M,M) did not remove the covariance but, in contrast to the other “drift” schemes, reversed its sign so that F_drift_ exceeded F_hom_, which is in accord with the hypothesis that it introduces an increased “cost” of moving toward the extremes compared to **G**_VR2_(M,M).

### Managing the Rates of Inbreeding

**FIGURE 4:**
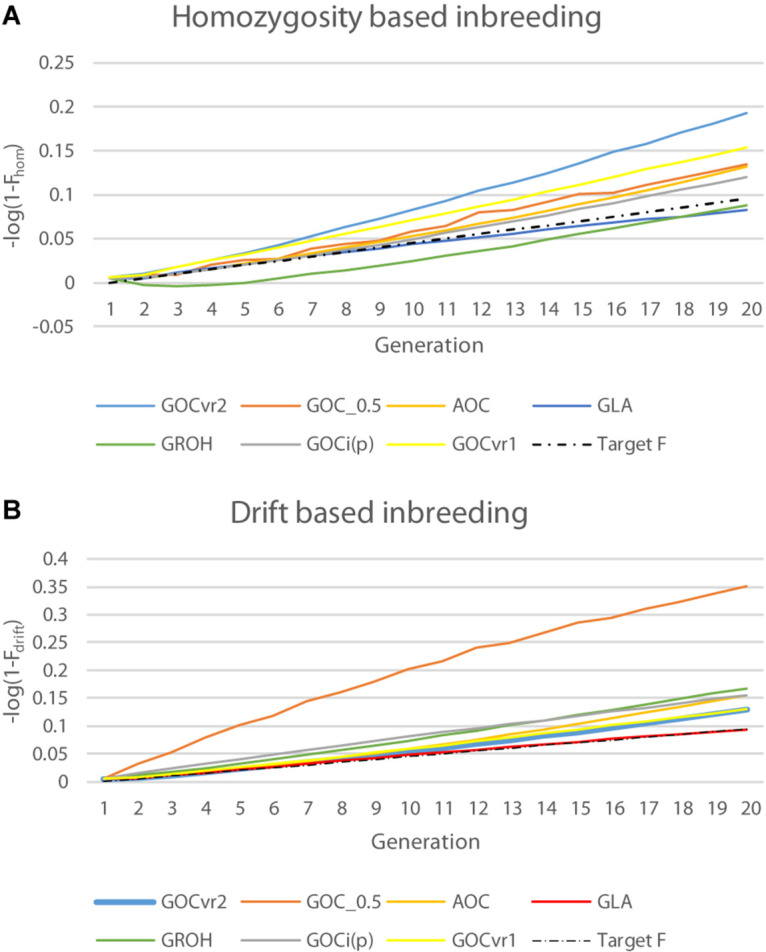
Changes in inbreeding coefficients *F*drift and *F*hom for the neutral loci of Panel N over time plotted on a logarithmic scale where a constant rate of inbreeding results in a linear increase of over time: **(A)** natural logarithm of (1–F_hom_); and **(B)** natural logarithm of (1–F_drift_).

Table 2 shows Δ*F*_drift_ and Δ*F*_hom_ for the different schemes for Panels M and N, and Figure 4 shows F_drift_ and F_hom_ over time. Figure 4 shows that log(1-*F*_drift_) is approximately linear with generation for all schemes, in contrast to log(1-*F*_hom_) where some schemes, e.g., **G**_ROH_(M,M) show marked curvilinearity.

For **G**_VR2_(M,M), Δ*F*_drift_ for Panel M was directly controlled and was on target at 0.005, but Δ*F*_hom_ was more than double this target, due to the covariance described above. For Panel N, Δ*F*_drift_ was greater and Δ*F*_hom_ was less than observed for Panel M, so the difference was less extreme. The increase in Δ*F*_drift_ was due to Panel N’s LD with QTL that was not accounted for by its LD with Panel M, while the decrease in Δ*F*_hom_was due to the allele frequencies for loci in Panel N being subject to weaker regulation due to their imperfect LD with those in Panel M. The same pattern of differences between Δ*F*_drift_ and Δ*F*_hom_was observed in a less extreme form with **G**_VR2_(∼,M) as here the imperfect LD between Panels M and N is still important but the more favored marker alleles in Panel M change randomly from generation to generation. The outcome for Δ*F*_drift_ shown in Table 2 for **G**_*VR1*_(M,M) for Panel M is greater than the target, as F_drift_ and F_hom_ weight all loci in a panel equally, whereas the management weights the drift by 2*p*_0,*k*_(1−*p*_0,*k*_), consequently the LD with QTL is more weakly constrained for loci with low MAF in Panel M, which is where the impact of the covariance is greatest (Figure 3). This also explains the lower Δ*F*_hom_ observed for **G**_*VR1*_(M,M). The results for **G**_*i(p)*_(M,M) shown in Table 2 reflect the changed sign in the covariance in that Δ*F*_hom_ was less than Δ*F*_drift_. Unlike **G**_VR2_(M,M), the constraint applied was only indirectly related to F_drift_ or F_hom_ and so the achieved rates were not expected to meet the target, although Δ*F*_hom_ was close to the target for Panel M.

As with **G**_*i(p)*_(M,M) the simulated management for the measures based on homozygosity, **G**_0.5_(M,M) and **G**_ROH_(M,M), did not explicitly control *F*_drift_ or *F*_hom_, However, Δ*F*_hom_ was close to the desired target for **G**_ROH_(M,M) when measured in both Panels M and N. **G**_ROH_(M,M) showed a curvilinear time trend for F_hom_ mainly due to a negative Δ*F*_hom_ during the first few generations, after which it increased with time and was rising faster than **G**_LA_(M,M) at the end of the period; in contrast Δ*F*_drift_ was approximately linear. The accelerating Δ*F*_hom_ maybe caused by ROHs failing to accumulate inbreeding as haplotypes recombine, so reducing the length of IBD segments below the thresholds implicit in ROH methods, while this older inbreeding is captured by *F*_hom_. To test this, the minimum length of a contributing ROH was halved to ∼3.5 from ∼7 Mb but results were nearly identical to those shown in Table 3 (result not shown). **G**_0.5_(M,M) has the highest *F*_drift_, because it explicitly promotes allele frequency changes to intermediate frequencies for all loci.

**TABLE 3:** Genetic gain (and its SE) after 20 generations of selection expressed in initial genetic standard deviation units, and inbreeding measured by homozygosity for Panel N of neutral loci at generation 20 for comparison.

Scheme	Gain	SE	*F*_hom_^1^	*F*_drift_^1^
**Drift measures**				
G_VR2_(M,M)	7.124	0.002	0.18	0.12
G_VR2_(M,D)	7.107	0.003	0.17	0.12
G_*VR1*_(M,M)	6.680	0.002	0.15	0.12
G_*i(p)*_(M,M)	7.111	0.003	0.11	0.14
**Homozygosity measures**				
G_0.5_(M,M)	6.734	0.004	0.13	0.30
G_ROH_(M,M)	9.099	0.003	0.08	0.15
**IBD measures**				
G_LA_(M,M)	7.188	0.002	0.08	0.09
**A**(M,∼)	9.890	0.003	0.12	0.14

*Scheme **G**_VR2_(_,M) is not shown as it was random selection. ^1^Standard errors <3 × 10^–3^.*

In contrast to all other schemes, Δ*F*_drift_ for **G**_LA_(M,M) was within 2% of the target for both Panels M and N (see Table 2) but was below target for Δ*F*_hom_ for both panels. The discrepancy for Δ*F*_hom_ is complicated by the dynamic pattern of the number of parents selected in this scheme (see Figure 2), which results in the expected heterozygosity being close to that for random mating in early generations, but ∼0.005 less than random mating in later generation as a result of the degree of non-random mating introduced by the smaller number of parents. Therefore estimating Δ*F*_hom_ from observed heterozygosity will underestimate the true value and explains a substantial part of the observed deviation from the target value of 0.005. Figure 4 shows **G**_LA_(M,M) was lowest for *F*_drift_ and *F*_hom_ in generation 20 with near constant rates. The results from AOC were qualitatively similar except that both Δ*F*_hom_ and Δ*F*_drift_ exceeded the target rates by 40% in both panels. This is due to the hitch-hiking of neutral loci with the changes in QTL frequencies arising from the LD generated within families and is unaccounted by using expectations of IBD based on pedigree.

### Genetic Gain

**FIGURE 5:**
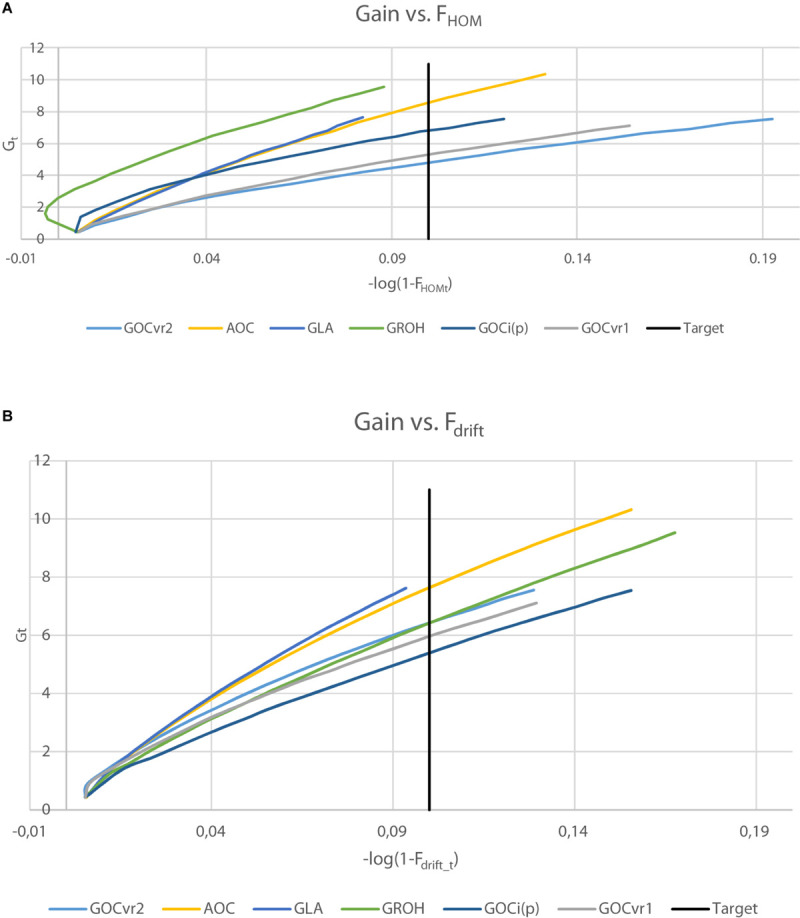
Genetic gain, *Gt* plotted against inbreeding for generations 1–20, where inbreeding is transformed to a logarithmic scale by –log(1-*F*_*t*_) for F_hom_ **(A)** or F_drift_ **(B)**. For ΔF = 0.005, the target after 20 generations is shown (–log(1-*F*_*t*_) = 0.1).

Table 3 shows the genetic gains of the schemes achieved after 20 generations of selection and Figure 5 shows the gain achieved over time as a function of *F*_drift_ and *F*_hom_ for the neutral markers in Panel N. Figure 5 allows comparisons to be made at the same *F*_drift_ or *F*_hom_ and offsets, in part, the unequal rates of inbreeding observed among the different schemes.

The genetic gains were very similar (within 0.3%) for the schemes **G**_VR2_(M,M) and **G**_VR2_(M,D) where the latter differs only in using a second marker panel for inbreeding management which was unambiguously neutral. Given the small difference in their inbreeding rate at the neutral loci in Panel N (Tables 2, 3), this indicates that separate panels of markers for gain and for diversity is unnecessary for such schemes. The **G**_LA_(M,M) scheme yielded significantly more genetic gain than **G**_VR2_(M,M), at lower *F*_drift_ and *F*_hom_. **G**_ROH_(M,M) and **A**(M,∼) yielded substantially more gain, but their *F*_drift_ was also higher. The **A**(M,∼) scheme yielded the highest genetic gain of all the schemes compared, but, compared to its closest competitors, **G**_LA_(M,M) and **G**_ROH_(M,M), it also yielded more *F*_drift_ and/or *F*_hom_.

It is clear from Figure 5 that the ranking of the schemes for achieved gain differs according to whether drift or homozygosity is considered: e.g., **G**_ROH_(M,M) and **G**_*i(p)*_(M,M) schemes yielded relatively high gains given *F*_hom_, but relatively low gains given *F*_drift_, whereas **G**_VR2_(M,M) schemes yielded opposite results with low gains for *F*_hom_ and relatively high for *F*_drift_. The gain for the **G**_ROH_(M,M) scheme in early generations was accompanied by negative *F*_hom_ (Figure 5A). **G**_LA_(M,M) and **A**(M,∼) schemes performed relatively well as shown in both plots of Figure 5, with **G**_LA_(M,M) schemes seeming to yield in both plots slightly more gain per unit of inbreeding than **A**(M,∼). Although, the **A**(M,∼) gain is high relative to its inbreeding, the inbreeding rates were substantially larger than the target rate (which can be seen from Figure 5 by the curves extending far beyond the target). The **G**_LA_(M,M) scheme achieves the target rate of inbreeding closely for Δ*F*_hom_ and Δ*F*_drift_ (Table 2), and simultaneously converts inbreeding efficiently into genetic gain. Moreover, when testing genetic gains in generation 20 of the **G**_LA_(M,M) schemes to interpolated gains at the same overall inbreeding (average of *F*_hom_ and *F*_drift_) of the **A**(M,∼) and **G**_ROH_(M,M) schemes, the **G**_LA_(M,M) scheme yielded the highest gain in 65, respectively, 62 out of 100 replicates; i.e., generation 20 gains of **G**_LA_(M,M) were significantly higher than those of **A**(M,∼) and **G**_ROH_(M,M) (*P* < 0.01) at the same averaged inbreeding level.

### Number of Parents

Figure 2 shows the number of selected parents across the generations and shows that the schemes that use IBD based relationship matrices (**A**, **G**_LA_) and **G**_ROH_ select most parents. The selected number of parents for **G**_ROH_(M,M) may be artificially large due to the additions to the leading diagonal of **G**_ROH_ (on average 8.7) to make it positive definite. This process made the **G**_ROH_ matrix diagonally dominant, and so reducing **c**’**G**_ROH_**c** is driven by selecting more parents in order to reduce the impact of these diagonal elements and not about avoiding the selection of related animals. Non-positive definite **G**_ROH_ matrices could be inverted to obtain optimal solutions **c**, but these yielded much too high rates of inbreeding (result not shown) probably because optimal contributions **c** were found that resulted in negative **c**’**G**_ROH_**c**, which does not make sense and inbreeding was high and positive. Schemes using matrices constructed by the methodology of VanRaden (2008) (**G**_*VR1*_, **G**_VR2_, **G**_*i(p)*_, and **G**_0.5_) select fewest parents, implying that they are able to select relatively less related parents by their respective measure, and differences in relationships are relatively large in their respective matrices. Comparing results from Table 2 and Figure 2 suggests that the selection of relatively few parents is achieved by making use of the opportunities to induce covariances between allele-frequency-changes and initial frequencies that these schemes offer, which in turn affect the frequencies of heterozygotes.

### Genetic Variance

**FIGURE 6:**
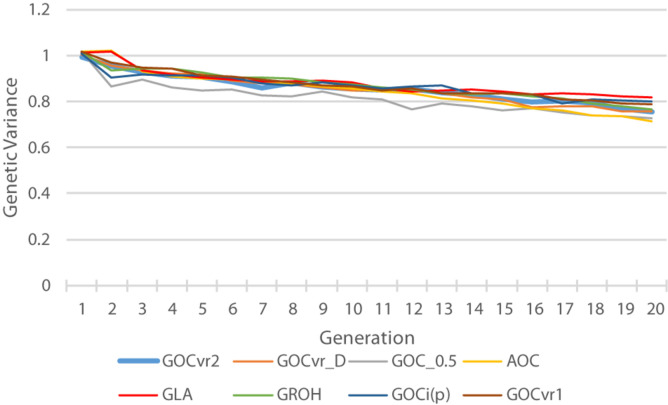
The trait genetic variance of the individuals plotted over time.

Figure 6 shows the genetic variance for the trait calculated from the true breeding values of the individuals. The **G**_0.5_(M,M) scheme loses substantial genetic variance at an early stage, and this relatively low genetic variance is maintained throughout the 20 generations of selection. Therefore striving for allele frequencies of 0.5 at the loci in Panel M does not maintain variation at the QTL in Panel Q, which is in accord with the results for Panel N in Table 2. The relatively low variance for **A(**M,∼) at generation 20 is a consequence of it relatively high genetic gain combined with its relative high rates of inbreeding. By generation 20, the **G**_LA_(M,M) scheme has lost least genetic variance, due to its rates of inbreeding not exceeding the target, and may explain why the **G**_LA_(M,M) scheme is very efficient in turning inbreeding into gain at the end of the selection period (Figure 5).

## Discussion

### Equivalence of Measures *F*_hom_ and *F*_drift_

In the classical work of Wright (1922) two natural measures of inbreeding were introduced concerned with the extent of drift on the one hand (here represented by *F*_drift_ and Δ*F*_drift_) and heterozygosity on the other (here represented by *F*_hom_ and Δ*F*_hom_), and in classical theory with neutral loci unlinked to QTL these perspectives were identical and directly linked to the occurrence of IBD. The results of this study show that these measures of inbreeding can differ substantially in genomic optimum contribution schemes even when there are no QTL in the genome [**G**_VR2_(∼,M); Table 2]. This is because the management in these schemes is commonly directed at the observed homozygosity or drift of the marker loci being monitored. For example, schemes that limit the rate of increase of homozygosity (as represented here by **G**_0.5_) induce a negative covariance between the change in allele frequency and the initial frequency, as an excess of minor alleles compared to classical expectations move toward intermediate levels. Conversely schemes managing drift and limiting changes in allele frequency (e.g., using *G*_*VR*2_) induce a positive covariance between change in allele frequency and the initial frequency, as an excess of minor alleles tend to move toward the nearest extreme. Consequently, systematic discrepancies occur between Δ*F*_drift_ and Δ*F*_hom_. These discrepancies are a property of the inbreeding management and not of selection *per se*, as they were unaffected by whether random GEBVs were used in the scheme or separate panels of SNPs were used for generating GEBV and management of inbreeding. In contrast to the management using the IBS allele frequencies of monitored markers, when IBD was used either via genomics information (*G_LA_*) or approximately (**A**, uninfluenced by markers) the equivalence of Δ*F*_drift_ and Δ*F*_hom_was re-established in the simulations, although not with *G*_ROH_ which is targeted toward IBD but is based on the homozygosity of haplotypes.

The origin of these covariances between allele frequency changes and initial frequencies can be seen when considering the form of the relationship matrix and is explored in detail in Supplementary Information 1. The negative covariance arising from *G*_0.5_ explicitly measures allele frequencies as deviations from 0.5, not from the base frequency *p*_0,*k*_ and consequently gains in this measure of diversity (but not necessarily IBD, as discussed later) are obtained by moving frequencies toward 0.5 offsetting any opposing changes prompted by selection objectives. The positive covariance, for example with *G*_*VR*2_, arises because drift of an allele to the more distant extreme is more heavily penalized compared to completely random drift as the GOC with *G*_*VR*2_ is constraining the square of the change. This will inevitably promote shifts to the nearest extreme, and more strongly so as *p*_0_ deviates more from ½. Since *G*_*VR*1_ is a re-weighting of the loci in *G*_*VR*2_ by *w*_*k*_/Σ_*locik*_*w*_*k*_ for locus *k*, where *w*_*k*_ = 2*p*_0,*k*_(1−*p*_0,*k*_), placing more weight on frequency changes for loci initially closer to ½, it would be expected the discrepancy between *F*_drift_ and *F*_hom_would be less for *G*_*VR*1_ than *G*_*VR*2_ as observed in the simulations (see Table 2 and Figure 4). Moving to management using the total intensity applied over time (*G*_*i*(*p*)_) penalizes deviations that move toward the extremes more heavily than those toward intermediate frequencies (as *di*/*dp* = [*p*(1−*p*)]^−1/2^; Liu and Woolliams, 2010), and this changed the sign of the discrepancy although its magnitude was decreased compared to *G*_*VR*2_.

*G*_*VR*2_, which was used by Sonesson et al. (2012), controlled Δ*F*_drift_and met the target for the panel used (see Table 2) but Δ*F*_hom_ was much greater due to the covariance discussed above. This agreed with the findings of de Beukelaer et al. (2017), where it was suggested that the covariance between change in frequency and its initial value could be the cause of this. However, these authors also reset the allele frequencies for the reference population in the **G**_*VR1*_ matrix every generation to the current generation frequencies, which implies that changes in allele frequency in each generation are constrained without reference to their accumulated change over earlier generations. In a continuous selection scheme, the allele frequency changes of successive generations are positively correlated; thus, although the variance of the change in allele frequency within a generation may have been on target, the variance of the cumulative allele frequency change over generations will exceed the target value due to these positive correlations, as observed in their study. This distinction in methodology will have affected all findings on GOC in the study of de Beukelaer et al. (2017).

Sonesson et al. (2012) found that *G*_*VR*2_ schemes achieved their target rate of inbreeding based on IBD using loci with 2N alleles scattered across the genome. Details of the founder populations used in their study were presented in Sonesson and Meuwissen (2009), which revealed that their SNP-BLUP marker panel was selected for intermediate frequencies in order to mimic a typical SNP-chip marker panel. This is very different from the SNP-BLUP panel used here which was a random sample of whole genome sequence data, and hence dominated by extreme allele frequencies (Figure 1). The strength of the covariance underlying the discrepancy between *F*_drift_ and *F*_hom_ depends on the distribution of $\left(p_{0}-\frac{1}{2}\right)$, and so in Sonesson et al. (2012) any discrepancy would have been much reduced. In the context of the current results, it was most similar to using *G*_*VR*1_ where the intermediate loci are more heavily weighted. Conclusions from these considerations are (i) that the discrepancies between the different measures of rates of inbreeding are extreme in WGS data, due to their extreme allele frequencies (Figure 1); and (ii) the discrepancies are a property of the panel used to manage diversity and not the remaining loci, as the IBD-alleles used by Sonesson et al. (2012) have low MAF by construction. Hence, for typical SNPs from chips, the discrepancies between *F*_drift_ and *F*_hom_ are expected to be present but smaller than those in Table 2.

### Management of Diversity

An important aspect of a tool to manage diversity is that it is predictable in meeting its targets, and this can be examined for the marker panel, for the unmanaged neutral markers, and for *F*_drift_ and *F*_hom_. In this respect, *G*_*VRn*_ meets the target but only for *F*_drift_ and only in the marker panel (i.e., not in the unmanaged panel) whereas *G*_*LA*_ meets the target (with only minor deviations) for both *F*_drift_ and *F*_hom_ for both panels. All others failed to meet the target rate to a greater or lesser degree and would need to be calibrated, possibly in every generation, to meet the targets set at neutral loci. In practice, this would require as realistic as possible simulations of the practical breeding scheme using the current situation as a starting point.

A key management objective in breeding schemes is the efficient generation of gain from the genetic variance in the objectives, and conserving the variation at the (currently) neutral loci, and here the IBD-related schemes were best when compared to F_drift_ or F_hom_ of neutral loci. On an average of F_drift_ and F_hom_, **G**_LA_ was more efficient than **G**_ROH_, which gave different rates for Δ*F*_hom_ and Δ*F*_drift_, would require regular calibration, and (in the current implementation following de Cara et al., 2013) always required very large number of parents, which in practice would usually demand additional scheme resources. Henryon et al. (2019) observed that using **A** appeared to be more efficient than using *G*_*VR*2_, and this was confirmed here. The differences between schemes using *G*_*LA*_ and **A** were small when plotted against *F*_drift_ or *F*_hom_ but the *G*_*LA*_ scheme was the only scheme tested here that combined high efficiency with rates of inbreeding close to and not exceeding the target rate of inbreeding of 0.005. This supports the conclusion of Sonesson et al. (2012) that genomic selection requires genomic control.

One consequence of entering the genomics era is that the meaning of diversity and its management in practice is more open to discussion, as the pedigree is no longer the only tool to measure and manage it. For example, the number of polymorphic loci could be used as a measure, which might underpin major concerns over the disappearance of known rare alleles in the scheme. Further, in the pedigree inbreeding framework, the measure used is the fraction of variance that is expected to have been lost from the reference base. In the genomic era, if the measure is simply defined as the genetic variance defined by IBS and maximized, there is scope for increasing diversity by the directional selection of loci toward intermediate frequencies as an objective. These measures have been explored elsewhere (see Howard et al., 2017 for a review). In general, attaching values (e.g., selection index weights) to genetic diversity is a very difficult task (e.g., Brisbane and Gibson, 1994; Wray and Goddard, 1994; Goddard, 2009; Jannink, 2010; Howard et al., 2017), which becomes especially clear in view of the aforementioned goals of diversity management, where diversity is required at many (hypothetical) traits simultaneously. Breeders have generally more of an idea about their target rate of inbreeding than on what weight to give to a diversity measure. Although the actual choice of the target rate of inbreeding remains somewhat arbitrary, guidelines have been developed over the years (Woolliams et al., 2015, for a review).

Here, it is argued that an over-riding objective for many populations such as livestock or zoo populations, beyond the breeding goals that underlie the selection on the EBV, is to manage over time the risks associated with the unmeasured attributes of a reference population (e.g., unrecognized deleterious recessives, drift in desirable holistic qualities, epistatic variance). In this respect, all approaches used in this study refer back directly to the established reference (base) population. As mentioned above, other perspectives may be advanced such as increasing the genetic variance at neutral loci by increasing heterozygosity (e.g., de Beukelaer et al., 2017). This could be achieved by the promotion of allele frequency changes toward intermediate values, as exemplified by *G*_0.5_ in this study, however, this raises issues that require further consideration. Firstly, changes in allele frequency result from multiple copies of a subset of base generation alleles, so increasing frequency is promoting IBD based inbreeding (it is analogous to changing QTL frequency). Secondly, if carried out with a marker panel, then increasing heterozygosity of the marker loci does not necessarily increase heterozygosity among unmonitored neutral loci, which is the objective. In these simulations, the near avoidance of overall loss of heterozygosity in the marker panel by GOC_0.5_ during selection was accompanied by much greater drift and more loss of heterozygosity in the unmonitored neutral loci than was achieved using IBD based inbreeding management. In contrast, the use of IBD in *G*_*LA*_ has information on the unobserved heterozygosity and drift across all the unmonitored genome positions. It remains only a hypothesis that the management of heterozygosity and drift using IBS might perform better than IBD when WGS sequence data is available, with or without selection, although some studies have considered its use (Eynard et al., 2015, 2016; Gómez-Romano et al., 2016). The question how to weigh *F*_hom_ and *F*_drift_ across all loci in the genome when a key objective is to manage unknown or unmonitored risks remains open.

While this study has focused on schemes where loss of genetic diversity is managed next to the maximization of genetic gain, other schemes may be pure conservation schemes, where no genetic change (gain) is desired, but the goals for genetic management are the same; i.e., conserve genetic variation, avoid inbreeding depression, avoid the occurrence of recessive diseases, and avoid random changes in phenotypic traits related to drift from a valued reference population. Strictly, with pure random selection, drift and homozygosity based inbreeding are expected to be the same [Eq. (2); and Falconer and Mackay, 1996]. However, minimisation of allele frequency changes or minimisation of loss of heterozygosity based on using IBS may still result in discrepancies between drift and homozygosity based inbreeding measures arising from the covariances described above. In fact, the potential covariance between the change in allele frequency and the initial frequency is expected to increase, since the inbreeding management term is more important in pure conservation schemes. This would also hold for GOC schemes with selection that aim for an *N*_*e*_ higher than our goal of *N*_*e*_ = 100. The greater potential for discrepancy argues for the use of IBD-based measures of relationship (*G*_*LA*_, or a more conservative use of **A**) to maintain diversity in such genetic conservation schemes.

The approach adopted here has not favored genetic variation at some neutral loci more than others *a priori*. Of course, a weighted genomic relationship matrix could be implemented and/or the multiple relationship matrices and associated constraints could be used to simultaneously control the genomic variation in different types of loci (Dagnachew and Meuwissen, 2016; Gómez-Romano et al., 2016). For example, a general **G** matrix covering the entire genome, and an additional **G** matrix controlling genetic diversity at e.g., the major histocompatibility complex, which is essential to the immune response of the animals. Alternatively, regions of the genome may be sought where average heterozygosity is to be increased (reduced) under the assumption that diversity is especially (or not) important in these regions. Regions with known recessive defects may be prioritized for diversity management, but direct inclusion of the known defects in the breeding goal seems more effective in controlling their frequencies. In practice, such regions with special emphasis for diversity management would need to be known *a priori*, and may only be effective if WGS was used for the relationships because, as shown here, what happens in a sample of loci does not necessarily predict what happens at loci outside that subset. Causative alleles of quantitative traits are quite evenly distributed across the genome (Wood et al., 2014), and as argued here the main goals of diversity management address many anonymous, unknown loci and hypothetical traits simultaneously, which makes it very hard to achieve a worthwhile prioritization of genomic regions for diversity management.

## Conclusion

• Contrary to classic inbreeding theory, inbreeding of unmanaged neutral loci as measured by drift (*F*_drift_) and by homozygosity (*F*_hom_) can differ very substantially, due to a covariance between the change in allele frequency and its initial frequency, leading to non-zero expected changes in frequency of a sign and magnitude determined by the initial frequency. Discrepancy between *F*_drift_ and *F*_hom_ occurs when inbreeding management is based on genomic relationship matrices (or similarity matrices) derived using IBS, but not when derived using IBD, which acts as a unifying concept for *F*_drift_ and *F*_hom_.
• The covariance generated is expected to be larger for WGS data where allele frequencies are extreme with typical MAF close to 0, than for SNP (chip) panels where allele frequencies are generally closer to ½.
• The (genomic) selection component of OC schemes does not cause the difference between *F*_drift_ and *F*_hom_.
• Using the same or a different panel for estimating GEBVs than for management of diversity in OC schemes makes only very small differences to genetic gain and the inbreeding in unmonitored neutral loci.
• Measures of genomic relationship can be classified as those based on changes in allele frequency change (e.g., **G**_VR2_) and directed at F_drift_; those based on homozygosity (e.g., **G**_0.5_) and directed at F_hom_; and IBD based (e.g., **G**_LA_); or combinations of these (e.g., **G**_ROH_). The choice of the relationship matrix depends very much on what objective it should serve.
• OC schemes that limit *F*_drift_ directly limit allele frequency changes, such as those using **G**_VR2_, result in low Δ*F*_drift_ at the expense of high Δ*F*_hom_. Schemes using **G**_*VR1*_ will be less extreme in this than **G**_VR2_.
• OC schemes that limit Δ*F*_hom_ (e.g., using **G**_0.5_), result in very low Δ*F*_hom_ at the expense of high Δ*F*_drift_ but both *F*_hom_ and *F*_drift_ may exceed targets at unmonitored neutral loci.
• The OC scheme using **G**_LA_, an IBD based relationship matrix, was the only scheme investigated here that managed homozygosity and drift based inbreeding within the target rate of 0.5%, yielding an effective population size ∼100; for all other schemes, either Δ*F*_drift_ or Δ*F*_hom_ or both exceeded their target.
• The OC scheme using **G**_LA_ yielded the highest gain per unit of inbreeding across both measures of inbreeding, closely followed by the scheme using **A**. The latter yielded high gain per unit of *F* but grossly exceeds target rates of inbreeding.
• The use of **G**_LA_ in practice requires the development of fast algorithms for its calculation.

## Data Availability Statement

The datasets generated for this study are available on request to the corresponding author.

## Author Contributions

TM contributed to study design, performed the simulations, and wrote the draft manuscript. AS developed the simulation software and contributed to discussions and the writing of the manuscript. GG contributed to discussions and the writing of the manuscript. JW contributed to study design, alternative schemes and methods, and discussions and writing of the manuscript. All authors approved the final version of the manuscript.

## Conflict of Interest

The authors declare that the research was conducted in the absence of any commercial or financial relationships that could be construed as a potential conflict of interest.
