# Supplementary material for: Management of Genetic Diversity in the Era of Genomics
Source: Front Genet. 2020 Aug 13;11:880. doi: 10.3389/fgene.2020.00880 (PMC7438563; doi:10.3389/fgene.2020.00880)
Supplement: Supplementary file 1 [file Data_Sheet_1.pdf]

Supplementary Information 1.

Covariance between allele-frequency changes and initial frequency causes $F_{hom} \neq F_{drift}$

We follow here the notation in the main text, i.e. allele frequency changes for locus *k* are $\delta p_{t,k} = p_{t,k} - p_{0,k}$ and follow a derivation similar to that of De Beukelaer et al. (2017) to show that the expectation from classical theory that $F_{hom} \neq F_{drift}$ does not hold when there is covariance between $\delta p_{t,k}$ and $p_{0,k}$.

The heterozygosity in generation *t*, for locus *k* is $H_{t,k}$:

$$\begin{aligned} H_{t,k} &= 2p_{t,k}\left(1-p_{t,k}\right) \\ &= 2(p_{0,k}+\delta p_{t,k}) - 2\left(p_{0,k}+\delta p_{t,k}\right)^2 \\ &= H_{0,k} - 2\delta p_{t,k}\left(2p_{0,k}-1\right) - 2\delta p_{t,k}^2 \end{aligned}$$

Therefore $H_{t,k}/H_{0,k} + \delta p_{t,k}^2/[p_{0,k}(1-p_{0,k})] = 1 - 2\delta p_{t,k}(p_{0,k} - \tfrac{1}{2})/[p_{0,k}(1-p_{0,k})]$

For equal weighting over loci, the first of these terms is the contribution of locus *k* to $1-F_{hom}$ and the second term is the contribution to F_{drift}. Therefore assuming that over all loci considered $E[\delta p_{t,k}] = 0$ which may be assumed for a sufficiently large sample of neutral loci with randomly chosen reference alleles, and taking expectations:

$$\begin{aligned} F_{hom} - F_{drift} &= 2\,\mathrm{cov}(\delta p_{t,k};(p_{0,k} - \tfrac{1}{2}))/[p_{0,k}(1-p_{0,k})] \\ &= 2\,\mathrm{cov}\left(\delta p_{t,k}\Big/\sqrt{p_{0,k}(1-p_{0,k})};\, p_{0,k}\Big/\sqrt{p_{0,k}(1-p_{0,k})}\right) \end{aligned} \qquad \text{[S1.1]}$$

Therefore the difference may be seen as twice the covariance of the change in allele frequency with deviation of the initial frequency from ½, with both scaled by $\sqrt{p_{0,k}(1-p_{0,k})}$.

Under random selection (random sampling of alleles), $E[\delta p_{t,k} \mid p_{0,k}] = 0$, so the covariance is 0, and the classical expectation $F_{hom} = F_{drift}$ holds. Here minor alleles with frequency p_0 ultimately drift to loss with probability $1-p_0$, i.e. most frequently, but the net covariance is zero due to the rarer fixation events arising from large frequency changes. For example, when $p_0 = 0.1$, the loss of the allele represents a shift of -1/3 of an initial s.d. for a fraction 0.9 of loci, whereas fixation represents a shift of 3 initial s.d, or 9-fold in the opposite direction, for 0.1 of the loci.

In Fisher's infinitesimal model, allele frequency changes under truncation selection and stabilising selection are expected to be infinitesimal as allelic effects must be $O(n^{-\frac{1}{2}})$ to maintain a finite variance as $n \to \infty$, where *n* is the number of QTL, and finite genetic change requires that $\delta p_{t,k}$ is also $O(n^{-\frac{1}{2}})$. Consequently the covariance of frequency changes with initial frequencies will $\to 0$ as $n \to \infty$.

Various forms of selection result in $\mathrm{cov}(\delta p_{t,k};(p_{0,k} - \tfrac{1}{2})) \neq 0$. For example, selection for increased heterozygosity has $E[\delta p_{t,k}] = 0$ as it promotes $p_{t,k} \to \tfrac{1}{2}$ with $\delta p_{t,k} < 0$ for $p_{0,k} > \tfrac{1}{2}$

and $\delta p_{t,k} < 0$ for $p_{0,k} < ½$ with changes expected to balance. The drift is increased measured as $\delta p_{t,k}^2$, since a selection pressure is being applied, resulting in a large positive F_{drift}; but $F_{hom} < 0$ since homozygosity decreases. This discrepancy between F_{hom} and F_{drift} is due to the expression of the negative $cov(\delta p_{t,k}; (p_{0,k} - 1/2))$ generated by the selection in Equation S1.1

An inbreeding management strategy might aim to reduce drift by preventing minor alleles from drifting to their opposite extreme frequency, i.e. selection is the reverse of selection for increased heterozygosity, so for the minor alleles $p_{t,k} \rightarrow 0$ if $p_{t,k} < ½$, and $p_{t,k} \rightarrow 1$ if $p_{t,k} > ½$, and thus $\text{cov}(\delta p_{t,k};(p_{0,k} - ½)) > 0$. Heterozygosity will decrease rapidly, resulting in high F_{hom} but the positive $\text{cov}(\delta p_{t,k};(p_{0,k} - ½))$ allows the possibility of only a small F_{drift} (S1.1). This may sound artificial, but when selection follows an optimal contribution algorithm which constrains the squared allele frequency changes e.g. $\mathbf{G}_{VR2}$, the algorithm will implement such a selection strategy to some degree in order to achieve its goals. Thus, selection schemes that cause deviations from the classical expectation $F_{hom} = F_{drift}$ are possible, especially when selections are aimed at the management of genetic variation.

Supplementary Material 2

Construction of genomic relationship matrices: $\mathbf{G}_{\mathbf{i(p)}}$, $\mathbf{G}_{\mathbf{LA}}$ and $\mathbf{G}_{\mathbf{ROH}}$

A. Construction of the $\mathbf{G}_{\mathbf{i(p)}}$ matrix

The $\mathbf{G}_{\mathbf{i(p)}}$ relationship matrix is based on the following notions:

1. GOC$_{\mathrm{VR2}}$ constrains the sum of squares of allele frequency changes where frequency changes are scaled by their initial standard deviation, i.e. $\Sigma_{loci\,k}\left[\delta p_{t,k} / \sqrt{2p_{0,k}\left(1-p_{0,k}\right)}\right]^2$ where $\delta p_{t,k}$ is the accumulated change at time *t* for locus *k* (Woolliams et al., 2015). The $\delta p_{t,k} / \sqrt{2p_{0,k}\left(1-p_{0,k}\right)}$ term can be interpreted as proportional to the intensity of selection applied as it is an allele frequency change divided by initial standard deviation).

2. The concept of selection intensities may be extended to random selection/sampling of parents, which results in random allele frequency changes. Changes in frequency are the result of forces applied which may be randomly directed (c.f. Brownian motion): the random drift of allele frequencies is due to the accumulation of such random intensities over generations.

3. In the infinitesimal model, directional selection increases ΔF as a function of the squared selection intensity applied each generation (Woolliams, Wray and Thompson, 1993).

4. Combining notions (1) to (3) suggests that GOC$_{\mathrm{VR2}}$ can be viewed as attempting to manage inbreeding by constraining sum of squares of selection intensities which are randomly directed.

5. Intensities are not linear on the scale of $\delta p_{t,k}$ but are linear on the transformed scale of the intensity function *i(p)* where $i\left(p\right)=\sqrt{2}\,\mathrm{arcsin}\left(1-2p\right)$, which can also be transformed into a function of arcsin($\sqrt{p}$), (Liu and Woolliams, 2010). Therefore the total intensity required to move an allele from frequency p_0 to p_t is $i\left(p_t\right)-i\left(p_0\right)$. This suggests that intensities should be constrained on the scale of $i\left(p\right)$.

Following Woolliams et al. (2015), at a time *t* GOC$_{\mathrm{VR2}}$ constrains the average relationship of the selected parents calculated as $\mathbf{G}_{VR2}=\mathbf{c}^T\mathbf{X}_t\mathbf{X}_t^T\mathbf{c}/N_{loc}$, where ***c*** are optimal contributions of animals to the next generation, $\mathbf{X}_t$ has element (*j*,*k*) equal to $2\delta p_{t,k}(j)/\sqrt{2p_{0,k}(1-p_{0,k})}=\sqrt{2}\tilde{i}_{j,k}$ for animal *j* at locus *k* where $\delta p_{t,k}(j)$ is the deviation of the allele frequency within animal *j* (which is 0, ½ or 1) from the initial frequency $p_{0,k}$, and $\tilde{i}_{j,k}=\delta p_{(j,k)}/\sqrt{p_{0,k}\left(1-p_{0,k}\right)}$ is an approximation of the intensity required to move the allele from $p_{0,k}$, to the frequency in animal *j* (0, ½ or 1). Hence, $\tilde{\mathbf{i}}_t=\mathbf{X}^T\mathbf{c}$ denotes a vector of average intensities of the parents, when the parents are selected as indicated by the contribution vector ***c***. It follows that $\mathbf{c}^T\mathbf{G}_{VR2}\mathbf{c}=2\tilde{\mathbf{i}}_t^T\tilde{\mathbf{i}}_t/N_{loc}$.

The following applies the same reasoning on the scale where the intensities are additive i.e. *i(p)*, such that the averaging of the intensities by the contribution vector ***c*** does not result in an approximation. To calculate the average intensity of the selected parents on the *i(p)* scale, we calculate the breeding values of all the animals for each locus *k* for *i(p)*. On the *i(p)* scale, allelic effects at any locus *k* on intensities are additive. The breeding values at a single locus are thus: $-2p_{t,k}a_k$, $(1-2p_{t,k})a_k$, and $2(1-p_{t,k})a_k$ for homozygous reference allele, heterozygous, and homozygous alternative allele genotypes, respectively, where a_k is the additive effect of the allele (Falconer and Mackay, 1996). The additive effect equals half the difference in intensity between alternative homozygotes and reference allele homozygotes, i.e.: $a_k = \left[i(1)-i(0)\right]/2 = -\pi/\sqrt{2}$ for all *k*.

To account for the average intensity of the current population, i.e. the change of the population mean from the original frequency $p_{0,k}$, $i(p_{t,k})-i(p_{0,k})$ was added to all breeding values. Putting these terms together, the average breeding value of animal *j* at locus *k* is:

$T_{j,k} = i(p_{t,k})-i(p_{0,k})+2(1-p_{t,k})(-\pi/\sqrt{2})$ for alternative allele homozygotes

$T_{j,k} = i(p_{t,k})-i(p_{0,k})+(1-2p_{t,k})(-\pi/\sqrt{2})$ for heterozygotes

$T_{j,k} = i(p_{t,k})-i(p_{0,k})-2p_{t,k}(-\pi/\sqrt{2})$ for reference allele homozygotes

which yields a matrix of breeding values **T**. The intensities in **T** can be averaged over the parents to obtain the expected average intensity of their offspring, **T'c.** In $\mathbf{G}_{i(p)}$(M,M) as in $\mathbf{G}_{VR2}$(M,M) the sum of squared intensities is constrained i.e. $\boldsymbol{c'G}_{i(p)}\boldsymbol{c} = \boldsymbol{c'TT'c}/N_{loc}$ and so $\boldsymbol{G}_{i(p)}$ is constructed as $\boldsymbol{G}_{i(p)} = \boldsymbol{TT'}/N_{loc}$.

B. Construction of the $\mathbf{G}_{LA}$ matrix

For the construction of $\mathbf{G}_{LA}$, haplotypes were constructed from 700 discrete windows of 10 adjacent SNPs across the genome, which was assumed to have been phased. For each window a local gametic relationship matrix was constructed, $\boldsymbol{GAM}_{LA}$, with 2 entries per individual for its paternal and maternal gamete). Construction followed the tabular method of Fernando and Grossman (1989):

1. Base generation individuals were assumed unrelated with an identity numerator relationship matrix of size $2N_{base}$ x $2N_{base}$, where N_{base} is the number of base animals ($2N_{base}$ number of base gametes).
2. For all individuals, sorted from old to young, $j = N_{base}+1, \ldots, N_{total}$:
   a. *j*'s paternal haplotype_(j_{pat}) is compared to its sire's paternal haplotype (s_{pat}) and maternal haplotype (s_{mat}):
      If($j_{pat} = s_{pat}$ & $j_{pat} \neq s_{mat}$) then $\boldsymbol{GAM}_{LA}(j_{pat}, :) = \boldsymbol{GAM}_{LA}(s_{pat}, :)$

If($j_{pat} \neq s_{pat}$ & $j_{pat} = s_{mat}$) then $\boldsymbol{GAM_{LA}}(j_{pat}, :) = \boldsymbol{GAM_{LA}}(s_{mat}, :)$

Otherwise: $\boldsymbol{GAM_{LA}}(j_{pat}, :) = (\boldsymbol{GAM_{LA}}(s_{pat}, :) + \boldsymbol{GAM_{LA}}(s_{mat}, :))/2$

b. *j*'s maternal haplotype (j_{mat}) is compared to its dam's paternal haplotype (d_{pat}) and maternal haplotype (d_{mat}).

If($j_{mat} = d_{pat}$ & $j_{mat} \neq d_{mat}$) then $\boldsymbol{GAM_{LA}}(j_{mat}, :) = \boldsymbol{GAM_{LA}}(d_{pat}, :)$

If($j_{mat} \neq d_{pat}$ & $j_{mat} = d_{mat}$) then $\boldsymbol{GAM_{LA}}(j_{mat}, :) = \boldsymbol{GAM_{LA}}(d_{mat}, :)$

Otherwise: $\boldsymbol{GAM_{LA}}(j_{mat}, :) = (\boldsymbol{GAM_{LA}}(d_{pat}, :) + \boldsymbol{GAM_{LA}}(d_{mat}, :))/2$

c. Columns of $\boldsymbol{GAM_{LA}}$ obtained from symmetry: $\boldsymbol{GAM_{LA}}(:, j_{pat}) = \boldsymbol{GAM_{LA}}(j_{pat}, :)$ and $\boldsymbol{GAM_{LA}}(:, j_{mat}) = \boldsymbol{GAM_{LA}}(j_{mat}, :)$.

d. Diagonal elements of $\boldsymbol{GAM_{LA}}$ are always 1.

3. Calculate $\overline{GAM_{LA}}$ as the average of all $\boldsymbol{GAM_{LA}}$ matrices over all 700 haplotype windows.
4. Calculate element (*i,j*) of $\mathbf{G}_{LA}$ as $\mathbf{G}_{LA}(i,j) = \sum_{g=1}^{2}\sum_{g=1}^{2}\overline{GAM_{LA}}(i_g, j_g)/2$ where i_g and j_g are the gametes carried by *i* and *j*.

The condition 'Otherwise' in Step 2 includes situations where a recombination occurred and the offspring haplotype is a novel recombined haplotype and a 50/50 inheritance is assumed here for simplicity instead of estimating the fraction of the novel haplotype that is paternally and maternally inherited. The condition 'Otherwise' may also denote the situation where the paternal, maternal and offspring haplotype are all identical, and the haplotypes thus do not contain information about paternal/maternal inheritance.

C. Construction of the $\mathbf{G}_{ROH}$ matrix

The method of De Cara et al. (2013) was followed for constructing $\mathbf{G}_{ROH}$. A ROH was defined as $\geq$50 consecutive, strictly homozygote SNP loci (i.e. no heterozygote). In these simulations 50 consecutive SNP loci span ~ 7 cM. Note that the ROH were defined by IBS alone and the genome was assumed to be phased.

1. The diagonal of $\mathbf{G}_{ROH}$ for individual *i* was set as $1+F_{ROH}(i_1, i_2)$ where $F_{ROH}(i_1, i_2)$ is F_{ROH} for *i* with gametes i_1 and i_2.

2. The off-diagonals (*i,j*) were calculated as: $\mathbf{G}_{ROH}(i,j) = \sum_{g=1}^{2}\sum_{g=1}^{2}F_{ROH}(i_g, j_g)/2$ where i_g and j_g are the gametes carried by *i* and *j* respectively; and $F_{ROH}(a,b)$ denotes the F_{ROH} of an animal consisting of gametes *a* and *b* as defined above. It is not necessary to know which gamete is paternal and which is maternal due to the symmetry in this formula, and the availability of phased genotype data suffices. This is unlike $\mathbf{G}_{LA}$ above, where gametic origin is important.

Supplementary Information 3.

Linkage analysis and IBD for neutral loci linked to QTL.

Consider the set of neutral loci across the genome that have the frequency of a randomly chosen reference allele p_0, in a population that will form the base of a selection scheme, although without the assumptions of an idealised base. These loci will have a range of linkage disequilibrium (LD) relationships with all the QTL contributing to the future selection, some positively and some negatively, arising by chance or from previous selection.

1. *Neutral loci over generations*. When sampling gametes to produce the next generation for a locus *k* from this set, the probability of sampling the reference allele will be $p_0 + \delta p_{1,k}$, for some $\delta p_{1,k}$ which will depend on the LD of locus *k* with the QTL. Providing the set of loci sampled is sufficiently large and reference alleles are randomly chosen, one may assume $E[\delta p_{1,k}] = 0$. With random mating, the distribution of reference alleles for locus *k* in the next generation will be described by independent binomial sampling with probability of success $p_0 + \delta p_{1,k}$. Over all loci the distribution of reference alleles will have mean probability of success p_0 but will have extra-binomial variation arising from the variation in δp_1 within the set of loci. The drift variance for this set in the next generation is $var(p_1) = E[p_1^2] - p_0^2$. The first term is the expected frequency of homozygosity with random mating. If f_k is the inbreeding coefficient traced by IBD at locus *k* (which is >0 with true random mating), the frequency of a homozygote is f_k x probability one allele sampled from base is the reference allele $+(1-f_k)$ x probability two alleles drawn from base are both the reference allele. Therefore, the consequence of the extra binomial variance from the LD gives the result:

$$var(p_1) = f_i E[p_0 + \delta p_{1,i}] + (1-f_i)E[(p_0 + \delta p_{1,i})^2] - p_0^2 = f_i p_0(1-p_0) + (1-f_i)\mathrm{var}(\delta p_{1,i})$$

Therefore, since the second term is >0, the drift with selection is no longer equal to that for neutral unlinked loci. In subsequent generations the set of neutral loci with initial frequency p_0 will have a range of frequencies, as a result of multiple copies passed to the next generation for some gametes but not for others, and consequently for locus *k* $\delta p_{t,k} \neq \delta p_{1,k}$ as new LD is created from the selection. While each generation is binomial with extra-binomial variation, the parameters for each locus are dynamic across generations.

2. *Tracking IBD over generations with LE in the base.* Suppose the base population is now assumed to be idealised: animals are unrelated, non-inbred and all loci are in linkage equilibrium (LE), specifically marker locus *k* and any QTL locus are in LE. Selection is for the *Q* allele at the QTL and against the *q* allele and the frequency of the *Q* allele has steadily been increasing to generation *t*. It is feasible to construct the linkage analysis relationship matrix from the base, $\mathbf{G_{LA}}$, so each allele at time *t* represents a gametic pathway tracking an IBD allele

from the base population to *t*. Suppose after selection at time *t*-1, the two gametes sampled for an offspring in generation *t* both carry *Q* alleles. As above:

(a) With probability f_k, the two gametes are IBD at locus *k* and so are copies of a single gamete traced back to the base population, as indicated by the **GLA** matrix, and the frequency of it being the reference is p_0 due to the assumed LE with the QTL in the base.

(b) The two gametes are not IBD at locus *k* with probability $(1-f_k)$, i.e. the alleles stem from two different gametes from the base, as indicated by the **GLA** matrix, although both carried the *Q* allele. The probability of drawing the two reference alleles is p_0 for the first, and is also p_0 for the second due to the assumption of LE with the *Q* allele in the base. The second binomial sampling is independent even if there is substantial LD between locus *i* and the QTL in generation *t*-1 generated by the selection, since this LD is created by sampling multiple copies of one or more gametes in the base. However **GLA** explicitly traces the gametes back to the base, accounting for these multiple copies in (a).

Therefore there is no additional positive term in the drift variance despite the LD generated from multiple copies of gametes and the selection: i.e. $E[p_{t,k}^2] = f_k p_0 + (1-f_k)p_0^2$ and $H_{t,k} = 2(1-f_k)p_0(1-p_0)$. Since the locus is neutral $E[p_{t,k}] = p_0$, and $\text{var}(p_{t,k}) = f_k p_0(1-p_0)$. The conclusion is that (i) all LD generated from the base generation onwards is accounted for in **GLA**, (ii) conditional on the information from **GLA** the sampling of gametes at generation *t*-1 can be treated as independent binomial sampling from the base generation.

3. *Tracking IBD over generations with LD in the base.* Now consider again that LD exists in the base so that for locus *k* in the set of neutral loci with frequency p_0, the probability of sampling the reference allele at time *t* will be $p_{t,k} = p_0 + \delta p_{t,k}$. Following the result of paragraph (2) above, with **GLA** the deviation $\delta p_{t,k}$ is an attribute of the base and not a function of the subsequent selection. For locus *k*, $E[p_{t,k}^2] = f_i(p_0 + \delta p_{t,k}) + (1-f_k)(p_0 + \delta p_{t,k})^2$ and $E[H_{t,k}] = 2(1-f_k)(p_0 + \delta p_{t,k})(1 - p_0 - \delta p_{t,k})$ where the expectations are assuming random mating for locus *k*. Summing $2E[p_{t,k}^2] + E[H_{t,k}] = 2(p_0 + \delta p_{t,k})$, which is the standard expression for the allele frequency at time *t*, and holds for all loci irrespective of any variation in f_k. For a sufficiently large subset of neutral loci with base frequency p_0, the hypothesis is that $E[\delta p_{t,k} \mid p_0] = 0$ will hold over the range $0 < p_0 < 1$ so $E[p_{t,k}] = p_0$ then:

$$
\begin{aligned}
& 2E[p_{t,k}^2] + E[H_{t,k}] = 2p_0 \\
& \Rightarrow 2(E[p_{t,k}^2] - p_0^2) + E[H_{t,k}] = 2p_0(1-p_0) \\
& \Rightarrow 2var(p_t) + E[H_{t,k}] = 2p_0(1-p_0) \\
& \Rightarrow var(p_t)/[p_0(1-p_0)] + E[H_{t,k}]/H_0 = 1 \\
& \Rightarrow F_{drift} = F_{hom}.
\end{aligned}
$$

This holds for each subset of loci over the range $0 < p_0 < 1$.

4. *Management with* $\mathbf{G_{LA}}$. The impact of using $\mathbf{G_{LA}}$ is to (i) associate segments of each gamete of each candidate with the corresponding segment of the gamete in the base generation of which it is a copy, and (ii) make available the information on the number and distribution of the multiple copies of base generation gametes. This information is used when managing ΔF with $\mathbf{G_{LA}}$ and is independent of the initial frequencies of markers, their frequencies at a subsequent time point, and the sharing of IBS among base generation gametes. For this reason the hypothesis $F_{drift} = F_{hom}$ is plausible for a sufficiently large sample of neutral markers.
